# Hypernatremia in patients with severe traumatic brain injury: a systematic review

**DOI:** 10.1186/2110-5820-3-35

**Published:** 2013-11-06

**Authors:** Leif Kolmodin, Mypinder S Sekhon, William R Henderson, Alexis F Turgeon, Donald EG Griesdale

**Affiliations:** 1 Department of Medicine, Division of Critical Care Medicine, University of British Columbia, Vancouver, BC, Canada; 2 Department of Anesthesia, Pharmacology and Therapeutics, University of British Columbia, Vancouver, BC, Canada; 3 Department of anesthesiology, Division of Critical Care, Université Laval, Québec City, QC, Canada; 4 Centre for Clinical Epidemiology and Evaluation, Vancouver Coastal Health Research Institute, Vancouver, BC, Canada; 5 Critical Care Medicine, Vancouver General Hospital, Room 2438, Jim Pattison Pavilion, 2nd Floor, 855 West 12th Avenue, Vancouver, BC V5Z 1 M9, Canada

**Keywords:** Traumatic brain injury, Hypernatremia, Hyperosmolar therapy, Hypertonic saline

## Abstract

**Background:**

Hypernatremia is common following traumatic brain injury (TBI) and occurs from a variety of mechanisms, including hyperosmotic fluids, limitation of free water, or diabetes insipidus. The purpose of this systematic review was to assess the relationship between hypernatremia and mortality in patients with TBI.

**Methods:**

We searched the following databases up to November 2012: MEDLINE, EMBASE, and CENTRAL. Using a combination of MeSH and text terms, we developed search filters for the concepts of hypernatremia and TBI and included studies that met the following criteria: (1) compared hypernatremia to normonatremia, (2) adult patients with TBI, (3) presented adjusted outcomes for mortality or complications.

**Results:**

Bibliographic and conference search yielded 1,152 citations and 11 abstracts, respectively. Sixty-five articles were selected for full-text review with 5 being included in our study. All were retrospective cohort studies totaling 5,594 (range 100–4,296) patients. There was marked between-study heterogeneity. The incidence of hypernatremia ranged between 16% and 40%. Use of hyperosmolar therapy was presented in three studies (range 14-85% of patients). Hypernatremia was associated with increased mortality across all four studies that presented this outcome. Only one study considered diabetes insipidus (DI) in their analysis where hypernatremia was associated with increased mortality in patients who did not receive DDAVP.

**Conclusions:**

Although hypernatremia was associated with increased mortality in the included studies, there was marked between-study heterogeneity. DI was a potential confounder in several studies. Considering these limitations, the clinical significance of hypernatremia in TBI is difficult to establish at this stage.

## Background

Each year in the United States, 1.4 million patients suffer a traumatic brain injury (TBI), of which 235,000 patients are hospitalized and 50,000 die [1]. More than 40% of survivors will experience long-term disabilities [2]. Despite advances in neurocritical care, nearly a third of patients admitted with a severe TBI die, and less than half have a favorable neurologic outcome [3-5].

Primary and secondary injuries combine to result in increased vascular permeability, cerebral edema, and elevated intracranial pressure (ICP) [6-8], which itself is a consistent predictor of poor outcome in patients with TBI [9,10]. As such, control of ICP remains a central tenant in the management of patients with TBI [11]. Hyperosmolar therapy in the form of mannitol or hypertonic saline (HTS) promotes egress of water from brain interstitium thereby lowering ICP [12]. Mannitol is the agent of choice for the management of increased ICP in neurocritically ill patients with significant cerebral edema [13]. However, concerns over postadministration intravascular volume depletion and renal failure have led to the emergence of HTS as a therapeutic option [14].

Clinical studies of patients with TBI have confirmed the ICP lowering effects of HTS [15-17]. HTS has been used as both a resuscitative agent with bolus administration [18], as well as a constant infusion to treat hyponatremia or to institute hypernatremia in patients with TBI. However, there are several concerns with ongoing infusion targeting hypernatremia with HTS. First, there is no established dose of HTS or target serum sodium when instituting therapy. Hyperosmolarity from hypernatremia works in areas of “normal brain” where the blood–brain barrier remains intact and can result in increased brain volume in contusional areas [19]. Furthermore, the brain accommodates to HTS-induced sustained hypernatremia by intracellular idiogenic osmoles accumulation, which raises brain water content, restores brain volume, and leads to rebound increased ICP [12,20]. Despite the use in patients with persistently elevated ICP [21], authors have questioned the benefits of hypernatremia in patients with TBI [22]. In this context, we conducted a systematic review to investigate the association of hypernatremia and mortality in patients with severe TBI.

## Methods

This article reports on our systematic review in accordance with the Preferred Reporting of Items for Systematic reviews and Meta-Analyses (PRISMA) statement [23].

### Search strategy

We systematically searched MEDLINE (1966 to November 30, 2012), EMBASE (1977 to November 30, 2012), and The Cochrane Central Register of Control Trials (CENTRAL) (1948 to November 30, 2012) for randomised and observational studies of hypernatremia and mortality in patients with traumatic brain injuries. Studies in all languages were considered for inclusion. We hand-searched abstracts of the following conferences from 2000 to present: *American Thoracic Society, American College of Chest physicians, American Association for the Surgery of Trauma, European Association of Neurosurgical Societies, Congress of Neurological Surgeons,* and the I*nternational Neurotrauma* Symposium. We hand-searched the bibliographies of included studies and those of relevant review articles.

Using a combination of exploded Medical Subject Heading (MeSH) terms and text words, we constructed search filters for MEDLINE review for the concepts “*TBI*” and “*hypernatremia*.” All terms within a filter were combined with the Boolean OR operator. The TBI filter contained the MeSH term *craniocerebral trauma* and text words: *closed head injury, closed head trauma, traumatic brain injury, brain injury, tbi* and *chi*. The hypernatremia filter contained the MeSH terms *“saline solution, hypertonic”* and *hypernatremia* and text words: *hypernatremia, hypertonic saline*. The TBI and hypernatremia filters were then combined using the Boolean AND operator. Similar search strategy was employed for EMBASE. The search strategies are presented in Additional file 1.

### Selection criteria, data abstraction, and quality assessment

Independently and in duplicate, two authors (LK and DG) screened all articles using the following inclusion criteria: (1) compared hypernatremia (Na >145 mEq/L) to normonatremia, (2) included adult patients with TBI, (3) point estimate and 95% confidence interval for mortality or complications, and (4) described adjustment for potential confounders.

The following data were abstracted: study design, patient inclusion and exclusion criteria, admission diagnosis, Glasgow Coma Scale (GCS), definition and exposure to hypernatremia, use of hyperosmolar therapy (mannitol or HTS), patients diagnosed with diabetes insipidus (DI), and unadjusted and adjusted point estimates, and 95% confidence intervals for mortality or complications.

Methodological quality and risk of bias was assessed using the Newcastle-Ottawa Scale (NOS) [24]. The NOS is a 9-point scale which evaluates included cohort studies on selection and comparability of cohorts and the assessment of outcome.

### Analytical plan

We had initially planned to present a pooled point estimate for mortality across studies. However, it became clear that there was marked between study heterogeneity, which precluded a meta-analysis.

## Results

### Systematic search

**Figure 1:**
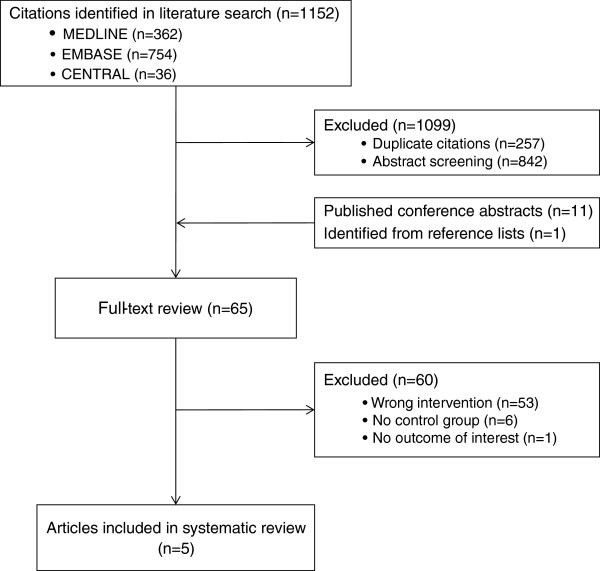
Study selection flowchart.

Bibliographic search yielded a total of 1,152 citations (Figure 1). We initially excluded 1,099 citations (257 duplicate citations and 842 from abstract screening). There were 11 abstracts identified from conference screening and one from reference screening. This resulted in 65 citations for full text review. Sixty abstracts were excluded (reasons listed in Figure 1), resulting in five studies included in our systematic review [25-29].

### Study characteristics and quality assessment

Study characteristics are presented in Table 1. Four studies were retrospective, observational cohorts with total of 5,594 (range 100–4,296) patients [25-28]. One study claimed to be a randomised trial but was likely an observational study as no description of randomization was provided, exposed and nonexposed groups were unequal, and a time-to-event analysis was performed to adjust for baseline risk [29]. Four studies were peer-reviewed full publications [25-28] and one was published in an abstract format [29]. There was marked between-study heterogeneity in terms of patient population, exposure to hypernatremia, consideration of DI in the analysis, and regression adjustment. Three studies included only patients with TBI [27-29], and two studies included patients with nontraumatic brain injuries [25,26]. Three studies included patients with a GCS ≤8 [26,28,29], whereas two studies did include patients notwithstanding the severity of the TBI [25,27]. The total scores for the Newcastle-Ottawa Scale are presented in Table 1 with further details presented in Additional file 2.

**Table 1:** Study characteristics included in systematic review

**Author, Yr**	**Inclusion & Patient Population**	**Admission GCS**	**Hyperosmolar therapy**	**Na levels**	**Outcome**	**Adjusted Regression Parameter**	**No. with DI (%)**	**NOS**
**(No. of patients)**				& **No. (%)**	& **No. (%)**			
Aiyagari [25] (n = 4296)	Admitted to ICU >17 yrs old TBI 17%	Median 13 (range 3 – 15)	Mannitol 605/4296 (14%); No HTS used	151 – 155 166/4296 (3.9%)	ICU mortality 33/166 (20%)	NR	DI due to brain death excluded; vasopressin use in 34/339 (10%) of pts. with hypernatremia	7
				156 – 160 78/4296 (1.8%)	ICU mortality 23/78 (30%)	NR		
				>160 95/4296 (2.2%)	ICU mortality 46/95 (48%)	OR 4.8^1^ (CI 2.4 – 9.6)		
						OR 4.2^2^ (CI: 1.8 – 10.6)		
Froelich [26] (n = 187)	GCS <9 & LOS ≥5d TBI 16%	NR	HTS as continuous infusion in 107/187 (57%)	> 155 69/187 (37%)	Creatinine >132.6 μmol/L 21/67 (31%)	OR 2.8 (CI: 1.3 – 6.2)	NR	8
					DVT 16/69 (23%)	OR 2.3 (CI: 0.9 – 5.9)		
					Infection 57/69 (83%)	OR 1.1 (CI: 0.4 – 2.9)		
Li [27] (n = 881)	TBI patients admitted to ICU ≥24 hrs	Median 7 (IQR 4 – 9)	Dose of mannitol reported, not proportion; HTS use NR	150 – 15434/881 (3.9%)	ICU mortality 7/34 (21%)	OR 9.5 (CI: 2.5 – 36.5)	6/34 (18%)	7
				155 – 159 66/881 (7.5%)	ICU mortality 28/66 (42%)	OR 4.3 (CI: 1.5 – 12.9)	27/66 (41%)	
				≥ 160 167/881 (19%)	ICU mortality 145/167 (87%)	OR 29.3 (CI: 11.5 – 74.4)	141/167 (84%)	
Maggiore [28] (n = 130)	TBI with GCS ≤8	Median 3 (range 3 – 8)	Mannitol in 64/130 (49%); HTS in 47/130 (36%)	>145 176/1103 (16%) patient days	14d mortality 34/130 (26%)	HR 4.2^3^ (CI: 1.6 – 10.2)	0/130	8
						HR 0.58^4^ (CI: 0.07 – 3.7)	25/130 (19%)	
Shehata [29] (n = 100)	TBI with GCS ≤8	Range 3 - 10	NR	>145 40/100 (40%)	ICU mortality 36/100 (36%)	HR 3.2 (CI: 1.78 – 5.7)	NR	3

GCS, Glasgow Coma Scale; Na, serum sodium; DI, diabetes insipidus; NOS, Newcastle-Ottawa Scale; ICU, intensive care unit; HTS, hypertonic saline; OR, odds ratio; CI, confidence interval; NR, not reported; DVT, deep vein thrombosis; TBI, traumatic brain injury; HR, hazard ratio; IQR, interquartile range.

Adjusted OR for mortality in patients who ^1^received mannitol and who ^2^did not receive mannitol.

Adjusted HR for mortality stratified on ^3^no DDAVP used and ^4^use of DDAVP.

### Hypernatremia and mortality

Four studies presented mortality data, either as mortality in the intensive care unit (ICU) [25,27,29] or 14-day mortality [28]. All studies demonstrated a significant association between hypernatremia and mortality. There were differential thresholds for hypernatremia in the studies that reported mortality, with two studies using Na >145 mEq/L [28,29], and two studies using >150 mEq/L [25,27]. The causes of hypernatremia were multifactorial and discussed below. Three studies included patients with DI [25,27,28], although this was only considered in the analysis in one [28]. One study with mortality as an outcome did not report data on diabetes insipidous [26,29].

In the study by Aiyagari and colleagues, which included both TBI and non TBI patients, hypernatremia was defined as Na >150 mEq/L with further stratification based on mild (151–155 mEq/L), moderate (156–160 mEq/L), or severe (>160 mEq/L) hypernatremia [25]. They observed a progressive increase in mortality in the univariate analysis from 59 of 458 (13%) for normonatremia (Na < 151 mEq) to 15 of 52 (29%) for mild, 13 of 39 (33%) for moderate, and 29 of 56 (52%) for severe hypernatremia, respectively. Thirty-four of 339 (10%) patients with hypernatremia received vasopressin (which may indicate DI), but this was not considered in the analyses. Finally, odds ratio (OR) for the association of serum sodium >160 mEq/L on ICU mortality adjusted for GCS, age, cerebrovascular disease, intracranial hemorrhage, and mechanical ventilation were presented. The adjusted odds ratio (OR) were stratified based on patients who received mannitol (OR 4.8, 95% confidence interval (CI): 2.4–9.6) and those who did not (OR 4.4, 95% CI: 1.8–10.6), although no formal test of homogeneity was performed. Similarly, Li and colleagues also observed an increased risk of ICU mortality with progressive increase in hypernatremia [27]. The final multivariable logistic regression model included GCS, APACHE II score, and dose of mannitol. There was a progressive increase in the proportion of patients who had DI from 6 of 34 (18%) in the mild hypernatremia group (Na 150–154 mEq/L), to 27 of 66 (41%) in the moderate hypernatremia group (Na 155–159), to 141 of 167 (84%) of patients in the severe hypernatremia group (Na ≥160 mEq/L). However, there was no consideration of DI in their analysis. Shehata and colleagues reported hypernatremia (Na >145 mEq/L) in 40 of 100 (40%) patients and an ICU mortality in 36% with a corresponding hazard ratio (HR) of 3.2 (95% CI: 1.8–5.7) after adjusting for baseline risk (not specified). Maggiore and colleagues performed a Cox proportional hazards analysis conditioning on days of ICU [28]. Hypernatremia (Na >145 mEq/L) occurred in 176 of 1,103 (16%) patient days. When adjusted for baseline risk of death and for each other, the use of DDAVP (HR 3.9, 95% CI: 1.4–10.3, *P* < 0.01), but not hypernatremia (HR 2.0, 95% CI: 0.81–4.8, *P* = 0.09), was associated with increased mortality. Point estimates stratified on desmopressin use also were presented (*P* = 0.06 for homogeneity), which resulted in improved model fit as assessed by a lower Bayes information criterion (BIC). Hypernatremia was associated with differential effects on mortality based on whether DDAVP was used or not. When DDAVP was used, hypernatremia was not associated with increased rate of 14-day mortality (HR 0.58, 95% CI: 0.07–3.7, *P* = 0.57). In contrast, in patients not exposed to DDAVP, hypernatremia was associated with increased rate of 14-day mortality (HR 4.2, 95% CI: 1.6-10.2, *P* = 0.004). Finally in the 51 (39%) patients in whom ICP was monitored, there was no association between hypernatremia and ICP.

### Hypernatremia and risk of complications

Only one study presented data on complications from hypernatremia [26]. This study compared patients who received a continuous 3% hypertonic saline infusion to normal saline in neurocritical care patients admitted for ≥5 days with an admission GCS <9. The authors observed an association between hypernatremia (Na >155 mEq/L) and an elevated serum creatinine >132.6 μmol/L (OR 2.8, 95% CI: 1.3–6.2, *P* = 0.01). This was an unadjusted analysis. There was no association between hypernatremia and the risk of deep vein thrombosis or risk of infection.

## Discussion

In this systematic review, we observed a consistent association of hypernatremia and mortality in patients with TBI. However, there also was heterogeneity in patient populations (case-mix), etiology and severity of hypernatremia, and the presentation of the data precluding a pooled analysis of data. In all but one study [28], DI (a known strong confounder) was either not reported or not considered in the analyses. Finally, one study on the complications associated with hypernatremia observed an increased risk of elevated creatinine [26].

Hypernatremia has been associated with increased mortality in a general ICU population [30,31], and in patients undergoing cardiac surgery [32]. However, it remains unclear whether the increased risk is attributable to the underlying medical condition of the patient or to the hypernatremia itself. This is further complicated in patients with TBI who have multiple additional reasons to develop hypernatremia, in particular renal loss of water (mannitol, DI) or hypertonic sodium gain [20]. Clinicians also may be reluctant to treat hypernatremia for fear of exacerbating cerebral edema and ICP. These factors expose a limitation of our systematic review and the included studies: confounding by indication. This bias exists when variables associated with outcomes in the study base also are associated with exposure [33]. In effect, patients will develop hypernatremia for a variety of different mechanisms, and some of these may be related to mortality. The decision to allow or induce hypernatremia by the treating clinician will be influenced by underlying patient characteristics. For example, clinicians may tolerate or induce progressive increase in serum sodium if the patients are thought to have increased ICP. Additionally, despite the hypothesized beneficial effects of reduced ICP from hypernatremia, any relationship between hypernatremia and ICP is strongly confounded due to the observational nature of the included studies. Thus, patients with more severe TBI who will more likely succumb from their injuries also may have higher degrees of hypernatremia. Although patients who have higher degrees of hypernatremia are at increased risk of dying, their prognosis is likely dictated by the underlying severity of illness, rather than the hypernatremia itself. Despite regression adjustment in the included studies, it would be insufficient to control for the strong confounding by the underlying severity of illness.

A good example of confounding by indication in the included studies was the management of patient with DI. DI is a risk factor for mortality in patients with TBI [34]. In one of the included study, DDAVP use was independently associated with an increased mortality [28]. Given these results, the authors appropriately provided subgroup point estimates for the association between hypernatremia and mortality in patients with and without DI (as indicated by DDAVP use). It must be noted that urine output or laboratory investigations were not used to support the presumption of DI in the studies. Despite this weakness, they observed that when DI was present, hypernatremia provided no additional attributable mortality. In contrast, in patients without DI, increased mortality was observed with hypernatremia. Two studies in this systematic review included patients with DI and did not adjust for this in the analyses [25,27]. Thus, it is likely that the mortality associated with hypernatremia observed in these studies may be partially explained by those patients who developed DI. For example, in the study by Li and colleagues, of the 167 patients who developed a serum sodium ≥160, 145 died (87%) and 141 (84%) had DI [27]. The effect of time is another strong confounder not addressed by the majority of studies included in this systematic review. Time as a covariate may either lead to bias or modify the relationship between serum sodium levels and ICP [35]. Use of Cox proportional hazards modeling, as used in the study by Maggiore and colleagues [28], allows for adjustment of time-varying covariates and reduces confounding by time. Finally, the association of hypernatremia and AKI seen in the study by Froelich and colleagues likely reflects unmeasured confounding as no adjusted analysis was presented [26].

There are additional important limitations to our systematic review. First, two studies included patients without TBI [25,26]. Thus, interpolating the overall results of these studies to the subgroup of patients with TBI is problematic. Second, although briefly discussed above, the underlying etiology for hypernatremia is not known. Hypernatremia may occur either through sodium gain (HTS) or free water loss (lack of access to free water, DI, or mannitol) [20,21]. It may be intentionally induced by the clinician (mannitol or HTS), tolerated (lack of free water), difficult to treat, or a combination of all of these. Importantly, the underlying mechanism and rational may be critical to interpreting the results of hypernatremia. The purpose of this systematic review was simply to highlight some of these important limitations. Finally, additional important consequences of induced hypernatremia from mannitol and HTS were not included: metabolic derangements, volume depletion, and overload.

There is a central question of great interest to neurointensivists. Namely, does hyperosmolar therapy by altering serum sodium concentration improve neurological outcomes after TBI? There is a clear paradox here that highlights a gap in the literature. On one hand, hyperosmolar therapy from mannitol or HTS has a strong biologic rationale and lowers ICP in randomised studies [12,15-17,36]. On the other hand, this systematic review raises concerns of hypernatremia, an endpoint for HTS therapy [21]. Authors have expressed concerns using HTS given the small size and nonrandomised nature of the available literature [22]. The dose, duration, and serum sodium target of the intervention remains unclear. Furthermore, HTS and mannitol have serious adverse effects on their own. Mannitol administration can lead to renal failure, metabolic derangements (hypochloremic alkalosis), and rebound increases in ICP. HTS can lead to volume expansion, heart failure, and hyperchloremic acidosis [12]. Although further observational studies may help to delineate a role for hypernatremia for the management of patients with TBI, whether or not a causality link exists with the use of therapies aiming to target high levels of natremia will need to be established.

## Conclusions

In our systematic review, we observed that hypernatremia in patients with TBI may be associated with increased mortality. However, the overall high risk of bias of included studies, the between-study heterogeneity in clinical design and analyses, and the remaining confounders may explain part of these findings. Further research should explore the impact of targeting lower versus higher levels of natremia in patients with traumatic brain injury.

## Competing interests

The authors declare that they have no competing interests.

## Authors’ contributions

LK was responsible for designing the study, bibliographic search, data abstract, data interpretation and drafting the manuscript. MS, WH and AF were responsbile for data interpretation and drafting the manuscript. DG was the principle investigator and responsible for the concept and design of the study. He performed the bibliographic search and data abstraction. He was involved in the interpretation of the data and drafting of the manuscript. All authors read and approved the final manuscript.

## Supplementary Material

Additional file 1 Search strategy used for MEDLINE and EMBASE to identify studies included in this systematic review. Click here for file

Additional file 2 **Newcastle-Ottawa Scale (NOS) **[24]** for included studies in systematic review.** Click here for file

## References

[B1] Langlois JA Rutland-Brown W Wald MM The epidemiology and impact of traumatic brain injury: a brief overview J Head Trauma Rehabil 2006 3 5 375 378 16983222 10.1097/00001199-200609000-00001

[B2] Selassie AW Zaloshnja E Langlois JA Miller T Jones P Steiner C Incidence of long-term disability following traumatic brain injury hospitalization, United States, 2003 J Head Trauma Rehabil 2008 3 2 123 131 18362766 10.1097/01.HTR.0000314531.30401.39

[B3] Myburgh JA Cooper DJ Finfer SR Epidemiology and 12-month outcomes from traumatic brain injury in Australia and New Zealand J Trauma 2008 3 4 854 862 18404048 10.1097/TA.0b013e3180340e77

[B4] Griesdale DEG McEwen J Kurth T Chittock DR External ventricular drains and mortality in patients with severe traumatic brain injury Can J Neurol Sci 2010 3 1 43 48 20169772 10.1017/s031716710000963x

[B5] Turgeon AF Lauzier F Simard J-F Mortality associated with withdrawal of life-sustaining therapy for patients with severe traumatic brain injury: a Canadian multicentre cohort study CMAJ 2011 3 14 1581 1588 21876014 10.1503/cmaj.101786 PMC3185074

[B6] Rosenfeld JV Maas AI Bragge P Morganti-Kossmann MC Manley GT Gruen RL Early management of severe traumatic brain injury Lancet 2012 3 9847 1088 1098 22998718 10.1016/S0140-6736(12)60864-2

[B7] Qureshi AI Suarez JI Use of hypertonic saline solutions in treatment of cerebral edema and intracranial hypertension Crit Care Med 2000 3 9 3301 3313 10.1097/00003246-200009000-00032 11008996

[B8] Chodobski A Zink BJ Szmydynger-Chodobska J Blood–brain barrier pathophysiology in traumatic brain injury Trans Stroke Res 2011 3 4 492 516 10.1007/s12975-011-0125-x PMC3268209 22299022

[B9] Lannoo E Van Rietvelde F Colardyn F Early predictors of mortality and morbidity after severe closed head injury J Neurotrauma 2000 3 5 403 414 10833059 10.1089/neu.2000.17.403

[B10] Marmarou A Anderson R Ward J Impact of ICP instability and hypotension on outcome in patients with severe head trauma J Neurosurg 1991 3 1s s57 s66

[B11] Bratton SL Chestnut RM Ghajar J Guidelines for the management of severe traumatic brain injury J Neurotrauma 2007 3 s1 s106 10.1089/neu.2007.9999 17511534

[B12] Ropper AH Hyperosmolar therapy for raised intracranial pressure N Engl J Med 2012 3 8 746 752 22913684 10.1056/NEJMct1206321

[B13] Foundation BT, Surgeons AA of N, Surgeons C of N Guidelines for the management of severe traumatic brain injury. I. Blood pressure and oxygenation J Neurotrauma 2007 3 Suppl 1 S7 S13 10.1089/neu.2007.9995 17511549

[B14] Himmelseher S Hypertonic saline solutions for treatment of intracranial hypertension Curr Opin Anaesthesiol 2007 3 5 414 426 17873594 10.1097/ACO.0b013e3282eff9ea

[B15] Vialet R Albanèse J Thomachot L Isovolume hypertonic solutes (sodium chloride or mannitol) in the treatment of refractory posttraumatic intracranial hypertension: 2 mL/kg 7.5% saline is more effective than 2 mL/kg 20% mannitol Crit Care Med 2003 3 6 1683 1687 12794404 10.1097/01.CCM.0000063268.91710.DF

[B16] Kamel H Navi BB Nakagawa K Hemphill JC Ko NU Hypertonic saline versus mannitol for the treatment of elevated intracranial pressure: a meta-analysis of randomized clinical trials Crit Care Med 2011 3 3 554 559 21242790 10.1097/CCM.0b013e318206b9be

[B17] Francony G Fauvage B Falcon D Equimolar doses of mannitol and hypertonic saline in the treatment of increased intracranial pressure Crit Care Med 2008 3 3 795 800 18209674 10.1097/CCM.0B013E3181643B41

[B18] Bulger E May S Brasel K Out-of-hospital hypertonic resuscitation following severe traumatic brain injury JAMA 2010 3 13 1455 1464 20924011 10.1001/jama.2010.1405 PMC3015143

[B19] Lescot T Degos V Zouaoui A Préteux F Coriat P Puybasset L Opposed effects of hypertonic saline on contusions and noncontused brain tissue in patients with severe traumatic brain injury Crit Care Med 2006 3 12 3029 3033 10.1097/01.CCM.0000243797.42346.64 16971850

[B20] Adrogué HJ Madias NE Hypernatremia N Engl J Med 2000 3 20 1493 1499 10816188 10.1056/NEJM200005183422006

[B21] Helmy A Vizcaychipi M Gupta A Traumatic brain injury: intensive care management Br J Anaesth 2007 3 1 32 42 10.1093/bja/aem139 17556349

[B22] Zygun D Sodium and brain injury: do we know what we are doing? Crit Care 2009 3 184 19804616 10.1186/cc8014 PMC2784343

[B23] Liberati A Altman DG Tetzlaff J The PRISMA statement for reporting systematic reviews and meta-analyses of studies that evaluate healthcare interventions: explanation and elaboration BMJ (Clinical research ed) 2009 3 b2700 10.1136/bmj.b2700 PMC2714672 19622552

[B24] Wells G Shea B O’Connell D The Newcastle-Ottawa Scale (NOS) for assessing the quality of nonrandomised studies in meta-analyses [Internet] 2009 [cited 2013 Jan 17]; Available from: http://www.ohri.ca/programs/clinical_epidemiology/oxford.asp

[B25] Aiyagari V Deibert E Diringer MN Hypernatremia in the neurologic intensive care unit: how high is too high? J Crit Care 2006 3 2 163 172 16769461 10.1016/j.jcrc.2005.10.002

[B26] Froelich M Ni Q Wess C Ougorets I Härtl R Continuous hypertonic saline therapy and the occurrence of complications in neurocritically ill patients Crit Care Med 2009 3 4 1433 1441 19242317 10.1097/CCM.0b013e31819c1933

[B27] Li M Hu YH Chen G Hypernatremia severity and the risk of death after traumatic brain injury 2012 Epub ahead of print 10.1016/j.injury.2012.05.021 22709549

[B28] Maggiore U Picetti E Antonucci E The relation between the incidence of hypernatremia and mortality in patients with severe traumatic brain injury Crit Care 2009 3 4 R110 19583864 10.1186/cc7953 PMC2750153

[B29] Shehata M Ragab D Khaled M Hegazy M Hussein A Khaled H Impact of hypernatremia on patients with severe traumatic brain injury Crit Care 2010 3 Suppl 1 355

[B30] Stelfox HT Ahmed SB Khandwala F Zygun D Shahpori R Laupland K The epidemiology of intensive care unit-acquired hyponatraemia and hypernatraemia in medical-surgical intensive care units Crit Care 2008 3 6 R162 19094227 10.1186/cc7162 PMC2646327

[B31] Lindner G Funk G-C Schwarz C Hypernatremia in the critically ill is an independent risk factor for mortality Am J Kidney Dis 2007 3 6 952 957 18037096 10.1053/j.ajkd.2007.08.016

[B32] Stelfox HT Ahmed SB Zygun D Khandwala F Laupland K Characterization of intensive care unit acquired hyponatremia and hypernatremia following cardiac surgery Can J Anesth 2010 3 7 650 658 20405264 10.1007/s12630-010-9309-1

[B33] Salas M Hofman A Stricker BH Confounding by indication: an example of variation in the use of epidemiologic terminology Am J Epidemiol 1999 3 11 981 983 10355372 10.1093/oxfordjournals.aje.a009758

[B34] Hadjizacharia P Beale EO Inaba K Chan LS Demetriades D Acute diabetes insipidus in severe head injury: a prospective study J Am Coll Surg 2008 3 4 477 484 10.1016/j.jamcollsurg.2008.04.017 18926448

[B35] Platt RW Schisterman EF Cole SR Time-modified confounding Am J Epidemiol 2009 3 6 687 694 19675141 10.1093/aje/kwp175 PMC2800260

[B36] Harutjunyan L Holz C Rieger A Menzel M Grond S Soukup J Efficiency of 7.2% hypertonic saline hydroxyethyl starch 200/0.5 versus mannitol 15% in the treatment of increased intracranial pressure in neurosurgical patients - a randomized clinical trial [ISRCTN62699180] Crit Care 2005 3 5 R530 R540 16277715 10.1186/cc3767 PMC1297608

