# Supplementary material for: Hypernatremia in patients with severe traumatic brain injury: a systematic review
Source: Ann Intensive Care. 2013 Nov 6;3:35. doi: 10.1186/2110-5820-3-35 (PMC3826846; doi:10.1186/2110-5820-3-35)
Supplement: Additional file 1 — Search strategy used for MEDLINE and EMBASE to identify studies included in this systematic review. [file 2110-5820-3-35-S1.doc]

Additional file 1: Search strategy used for MEDLINE and EMBASE to identify studies included in this systematic review.

**MEDLINE (Pubmed)**

1. Craniocerebral trauma/
2. Closed head injury.tw
3. Closed head trauma.tw
4. Traumatic brain injury.tw
5. Brain injury.tw
6. TBI.tw
7. CHI.tw
8. 1 or 2 or 3 or 4 or 5 or 6 or 7.
9. Saline solution, hypertonic/
10. Hypernatremia/
11. Hypernatremia.tw
12. Hypertonic saline.tw
13. 9 or 10 or 11 or 12
14. 8 and 13

**EMBASE**

1. Craniocerebral trauma/
2. Closed head injury.tw
3. Closed head trauma.tw
4. Traumatic brain injury.tw
5. Brain injury.tw
6. TBI.tw
7. CHI.tw
8. 1 or 2 or 3 or 4 or 5 or 6 or 7.
9. Sodium chloride/
10. Hypernatremia/
11. Hypernatremia.tw
12. Hypertonic saline.tw
13. 9 or 10 or 11 or 12
14. 8 and 13
