# Supplementary material for: Hypernatremia in patients with severe traumatic brain injury: a systematic review
Source: Ann Intensive Care. 2013 Nov 6;3:35. doi: 10.1186/2110-5820-3-35 (PMC3826846; doi:10.1186/2110-5820-3-35)
Supplement: Additional file 2 — Newcastle-Ottawa Scale (NOS) [24] for included studies in systematic review. [file 2110-5820-3-35-S2.docx]

| Author, Yr  (No. of patients) | Selection | Comparability | Outcome | Total Score |
| --- | --- | --- | --- | --- |
| Aiyagari, 2006^25^  (n=4296) | XXX | XX | XX | 7 |
| Froelich, 2009^26^  (n=187) | XXXX | XX | XX | 8 |
| Li, 2012^27^  (n=88 | XXX | XX | XX | 7 |
| Maggiore, 2009^28^  (n=13) | XXXX | XX | XX | 8 |
| Shehata, 2010^29^  (n=100) | X | X | X | 3 |
| The NOS comprises 3 categories (selection, comparability and outcome) with 9 items. Each item is scored as yes / no. The score for each item is represented by an X in the table above. | | | | |

Additional file 2: Newcastle-Ottawa Scale (NOS)^24^ for included studies in systematic review.
